# Effects of lovastatin treatment on the metabolic distributions in the Han:SPRD rat model of polycystic kidney disease

**DOI:** 10.1186/1471-2369-14-165

**Published:** 2013-07-31

**Authors:** Jelena Klawitter, Iram Zafar, Jost Klawitter, Alexander T Pennington, Jacek Klepacki, Berenice Y Gitomer, Robert W Schrier, Uwe Christians, Charles L Edelstein

**Affiliations:** 1 Department of Anesthesiology, University of Colorado, Aurora, CO, USA; 2 Division of Renal Diseases and Hypertension, University of Colorado Denver, iC42 Clinical Research and Development, 1999 North Fitzsimons Parkway, Bioscience East, Suite 100, Aurora, CO 80045-7503, USA

**Keywords:** PKD Han:SPRD rat model, Lovastatin, Biomarkers, Inflammation, Endothelial dysfunction

## Abstract

**Background:**

We previously demonstrated that lovastatin decreases cyst volume and improves kidney function in the Han:SPRD (Cy/+) rat model of ADPKD. Since endothelial dysfunction and inflammatory activity are evident in patients with ADPKD, we investigated whether lovastatin reduces the inflammation and vascular dysfunction and improves kidney cell energy metabolism of Cy/+ rats.

**Methods:**

Cy/+ and normal littermate control animals (+/+) were treated with either lovastatin (4 mg/kg/day) or vehicle (ethanol) from 3–8 weeks of age. ^1^H-NMR analysis was performed on water-soluble and lipid kidney fractions following perchloric acid extraction. Targeted liquid chromatography-tandem mass spectrometry (LC-MS/MS) was used to assess endothelial dysfunction, oxidative stress and inflammation markers in plasma and kidney tissue extracts.

**Results:**

Cy/+ rats showed perturbations in fatty acid metabolism and increased synthesis of pro-inflammatory lipoxygenases-produced bioactive lipids was observed. Lovastatin decreased inflammatory markers, specifically 13-HODE, 12-HETE and leukotriene B4. In Cy/+ rats, lovastatin reduced the elevated homocysteine and allantoin plasma levels and increased arginine, that is known to positively affect NO production.

In terms of kidney cell metabolism, Cy/+ rats showed reduced Krebs cycle activity. Treatment with lovastatin increased the Krebs cycle activity as well as the glycolytical lactate production, thus improving the overall energy state of the cystic kidney.

**Conclusion:**

As previously described, lovastatin was able to decrease kidney weight and cyst volume density in Cy/+ rats. The decrease in cyst volume was accompanied by a reduction in arachidonic acid-mediated inflammation markers, the normalization of metabolism of NO precursors and the improvement of kidney energy cell metabolism.

## Background

Autosomal dominant polycystic kidney disease (ADPKD) is the most common life-threatening hereditary renal disease, affecting approximately 1 in 200–400 individuals [1]. ADPKD is responsible for approximately 4% of end-stage renal disease (ESRD) in the United States and 8-10% in Europe [1]. The condition is characterized by progressive development of kidney cysts with renal enlargement and associated loss of renal function, such that approximately half of patients with ADPKD develop ESRD by 60 years of age [2].

Recent clinical studies have demonstrated the efficacy of statins in ameliorating progressive nephropathy [3-5]. Statins are known to improve renal blood flow [3,6] and glomerular filtration rate (GFR) by increasing single nephron GFR [7]. Statin treatment is also associated with enhanced vascular and glomerular nitric oxide production and attenuation of vascular inflammation [8].

Of particular relevance to ADPKD, statins have been shown to be beneficial in experimental angiotensin (Ang) II-mediated renal injury. Specifically, rats transgenic for human renin and angiotensinogen develop early hypertension, cardiac hypertrophy, and renal damage, with marked albuminuria and focal cortical necrosis [9]. Treatment of these rats with cerivastatin was associated with significant reductions in systolic blood pressure, serum creatinine, and albuminuria. Cerivastatin treatment was also associated with diminished inflammation as assessed by intercellular adhesion molecule-1 and vascular cell adhesion molecule-1 expression and renal infiltration with neutrophils or monocytes.

Beneficial effects of statin therapy on renal disease have also been observed in humans. Post-hoc subgroup analysis of the CARE study, a randomized trial of pravastatin vs. placebo, demonstrated that statin treatment significantly reduced the rate of decline of renal function in patients with moderate to severe chronic renal insufficiency, particularly in those with proteinuria [10,11]. In an open label study, atorvastatin administered with angiotensin-converting-enzyme inhibitor (ACEI) was also associated with decreased progression of renal disease [5]. These beneficial effects have been attributed to modulation of glomerular mesangial and interstitial inflammation and are independent of reduction in lipid concentrations.

The effect of statin treatment on renal function and structure has previously been studied in ADPKD. Specifically, van Dijk et al. have demonstrated increases in renal blood flow and GFR in response to a 4-week period of simvastatin treatment in 10 normocholesterolemic, normotensive patients with ADPKD [6]. It has also been proposed that statins may mediate progression of structural disease in ADPKD. In this regard, lovastatin has been shown in male Cy/+ rats to reduce the severity of PKD as assessed by kidney size, volume density of cysts, and blood urea nitrogen (BUN) concentration [12].

Although the underlying mechanisms are not well understood, it is believed that the renoprotective effects of statins are mediated by inhibition of G-proteins with resultant decreased cell proliferation. In addition, we have reported that statin therapy offers structural and functional benefits, including increased renal blood flow (RBF) and decreased BUN, independent of a change in mean arterial pressure (MAP), while ACE inhibitor therapy demonstrated structural benefit in association with a decrease in MAP [13].

Based on this knowledge, we hypothesized that, in addition to reducing the renal cyst volume, statins also a) reduce the inflammation and resulting vascular dysfunction and b) improve kidney cell energy metabolism in Cy/+ rats with PKD. To test our hypothesis we analyzed a comprehensive panel of endothelial dysfunction and inflammation markers in plasma and kidney tissue samples of Cy/+ rats treated with lovastatin for five weeks compared to normal control rats. In addition, a ^1^H-NMR-based metabolomics strategy was used to study changes in kidney cell energy metabolism as a result of PKD development and following lovastatin therapy of PKD rats.

## Methods

### Animals

The study was conducted in male heterozygous (Cy/+) and normal littermate control (+/+) Han:SPRD rats. The male Cy/+ rat develops clinically detectable polycystic kidney disease by 8 weeks of age, as evidenced by a doubling of kidney size and kidney failure compared with +/+ control rats [14,15]. A colony of Han:SPRD rats was established in our animal care facility from a litter that was obtained from the Polycystic Kidney Program at the University of Kansas Medical Center.

All animal protocols were approved by the University of Colorado Institutional Animal Care and Use Committee, and animal care was in accordance with the National Institutes of Health guidelines for ethical animal research (NIH publication No. 80–123). All animals were housed in cages in a temperature and light-controlled environment with free access to tap water and standard chow *ad libitum*.

### Experimental protocol

Male Cy/+ and +/+ rats were weaned at 3 weeks of age. The Cy/+ rats were treated with either lovastatin (4 mg/kg/day sc) or vehicle (100% ethanol sc, volume equal to lovastatin-treated group) for 5 weeks from 3 to 8 weeks of age. Water intake was monitored. The mean water intake for each rat for the 5-wk duration of the study was 817 ± 19 ml.

Lovastatin was obtained from Merck Sharp & Dohme Research Laboratories (Rahway, NJ). The dose of lovastatin was based on rat studies in ADPKD [12] and experimental nephrotic syndrome [16]. At the end of the study period, rats were anesthetized by an intraperitoneal injection of pentobarbital sodium (50 mg/kg body wt) and subjected to blood pressure and renal blood flow (RBF) studies before the kidneys were removed and weighed. The left kidney was fixed in 4% paraformaldehyde in PBS for 120 min and then put into 70% ethanol and embedded in paraffin for histological examinations. The right kidney was frozen at −70°C and used for metabolic analyses. EDTA plasma was collected as well and kept frozen at −70°C prior to analysis.

### Analysis of bioactive lipid mediators

Bioactive lipid mediators were analyzed using a high- performance liquid chromatography (HPLC)-tandem mass spectrometry (MS/MS) assay that was a modification of a previously described method [17]. Briefly, to 200 μL of EDTA plasma, 800 μL of internal standard containing methanol/ZnSO_4_ (70:30, v/v) solution (2 ng/mL mixture of internal standards, see below) were added. The homogenized kidney tissue (approximately 100 mg) was extracted in 1 mL of methanol/acetonitrile (1:1 v/v) and as previously described for plasma, 200 μL of extract was combined with 800 μL of internal standard containing methanol/ZnSO_4_ (70:30, v/v) solution (2 ng/mL mixture of internal standards, see below). The samples were vortexed for 10 minutes, centrifuged for 10 minutes at 13,000 g and transferred into a HPLC vial.

Fifty μL of the supernatant was injected into an online extraction LC-MS/MS system. The analytical column and solvents as described by Masoodi et. al. [17] were used. An API5000 mass spectrometer (AB Sciex, Concord, ON) was run in the positive electrospray ionization mode (ESI). The following hydroxyl fatty acids were quantified using the previously described MRM transitions [17]: hydroxy-octadecadienoic acids (9-HODE and 13-HODE), hydroxy-eicosapentaenoic acids (5-HEPE, 8-HEPE, 9-HEPE, 12-HEPE, 15-HEPE), hydroxy-eicosatetraenoic acids (5-HETE, 8-HEYE, 9-HETE, 12-HETE, 15-HETE, 20-HETE), 17-hydroxy-docosahexaenoic acid (17*S*-HDHA), resolving D1 (RvD1) and leukotriene B4 (LtB4). All compounds including the internal standards were purchased from Cayman Chemicals (Ann Arbor, MI).

*Endothelial dysfunction markers* including adenosine, arginine (Arg), asymmetric and symmetric dimethylarginine (ADMA and SDMA), cysteine (Cys), glutathione, homocysteine (Hcy), methionine (Met), S-adenosylhomocysteine (SAH) and S-adenosylmethionine (SAM) were quantified in EDTA plasma using a validated high-performance liquid chromatography-mass spectrometry (HPLC-MS/MS) assay. For more details, please refer to the Additional file 1.

The API5000 mass spectrometer (AB Sciex, Concord, ON) was run in the positive ESI mode using MRM. The following mass transitions were used (mass/charge, m/z): adenosine: 268.1 → 136.1; Arg: 175.2 → 70.1; ADMA: 203.2 → 46.2; SDMA: 203.2 → 172.2; d7-ADMA (internal standard): 210.2 → 77.2; cystine: 122.0 → 75.9; d2-cysteine (internal standard): 124.0 → 77.9; Hcy: 136.1 → 90.1; d4-Hcy (internal standard): 140.1 → 94.1; Met: 150.1 → 104.0; d3-Met (internal standard): 153.1 → 107.0; glutathione: 308.0 → 179.0; S-methylglutathione: 322.0 → 176.0; SAM: 399.0 → 250.1; d3-SAM (internal standard): 402.0 → 136.2; SAH: 385.0 → 136.2 and d5-SAH (internal standard): 391.0 → 137.2.

### ^1^H-NMR spectroscopy

^1^H-NMR analysis of kidney tissue samples was performed using a Varian INOVA NMR 500 MHz spectrometer equipped with 5-mm HCN-PFG probe (Varian, Palo Alto, CA). Following perchloric acid (PCA) extraction allowing for water-soluble and lipid fraction separation [18], lyophilized extracts were re-dissolved in 0.5 mL D_2_O and 1 mL of deuterated methanol/chloroform mixture (2:1, v:v) for water-soluble or lipid fraction extracts, respectively. The pH was adjusted to 7.2 with NaOD and DCl. TMSP (0 ppm, trimethylsilyl propionic-2,2,3,3,-d4 acid dissolved in D_2_O to 50 mmol/L) was used for metabolite chemical shift assignment (0 ppm) and quantification [18]. To suppress water in the extracts, a standard Varian pre-saturation sequence was used. ^1^H-NMR spectra were recorded at 500 MHz using spectral width of 12 ppm and 32 K data arrays, and 64 scans with 90° flip angle. The D1 time was 14.8 sec, which was required to fully relax all protons in the samples including the TMSP protons. Data analysis of the NMR data was performed using the TopSpin software (Bruker, Rheinstetten, Germany). Drift correction, zero filling from 32 K to 64 K data points and a Gaussian window function were applied to the free induction decay (FID) prior to Fourier transformation. Prior to integration, all ^1^H-NMR spectra were manually corrected for phase and baseline distortions. All NMR experiments were performed at the Systems Biology University of Colorado Cancer Center Core.

### Statistical analysis

All numerical data is presented as means ± standard deviation. Analysis of variance (ANOVA) followed by the Tukey *post-hoc* test was used to test for group differences. The significance level was p < 0.05 for all tests. SPSS statistics was used (version 19, IBM/SPSS, Chicago, IL).

## Results

### Fatty acid metabolism

**Figure 1:**
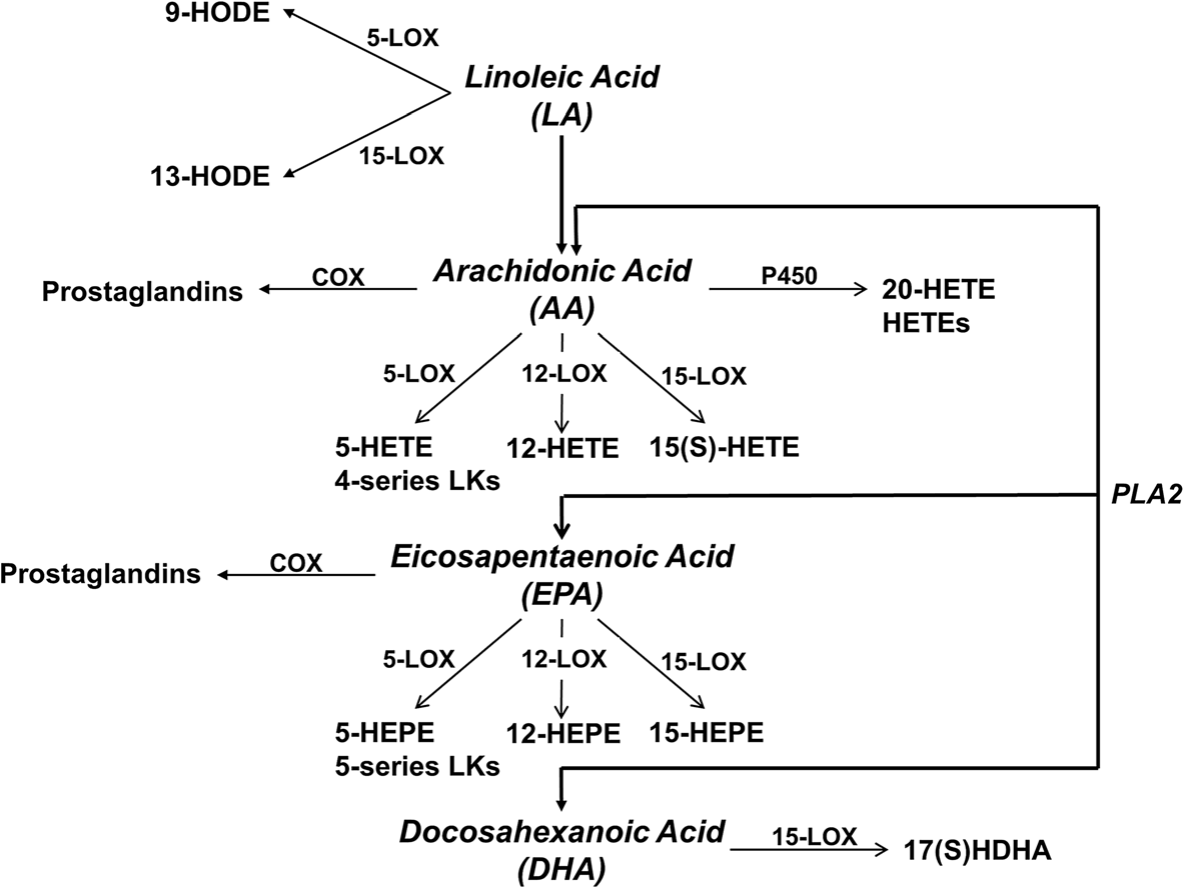
**Fatty acid metabolism.** Linoleic acid is metabolized by 5-LOX to 9-HODE, and by 15-LOX to 13-HODE. Arachidonic acid can be metabolized by COX enzymes to prostaglandins; through 5-LOX and 12/15-LOX enzymes into 4-series leukotrienes (LK) and HETE compounds: 5-HETE, 12-HETE, 15-HETE, 11-HETE, 9-HETE and 8-HETE; and by cytochrome P450 enzymes to HETEs including 20-HETE. Eicosapentaenoic acid is metabolized to HEPE compounds, mainly 5-HEPE, 12-HEPE and 15-HEPE. Docosahexanoic acid can be metabolized by 15-LOX to 17(S)HDHA.

Pathways of fatty acid metabolism considered in the present study are shown in Figure 1.

#### Linoleic acid (LA) metabolism

**Figure 2:**
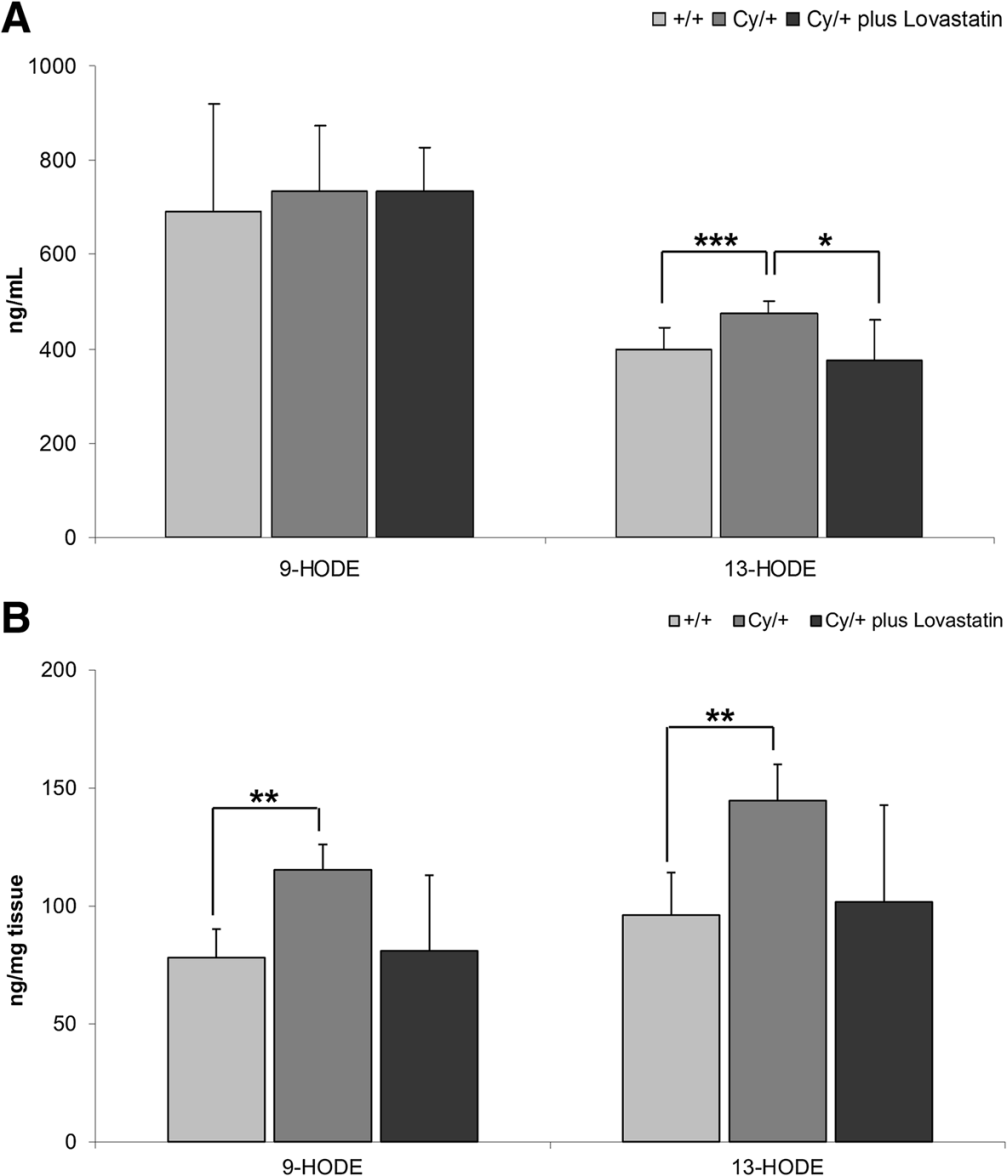
**Changes in pro-inflammatory lipoxygenases-mediated linoleic acid metabolites 9-HODE and 13-HODE in (A) plasma and (B) kidney tissue of healthy (+/+) and Cy/+ rats.** Cy/+ rats were additionally treated with 4 mg/kg/day lovastatin for 5 weeks. 9-HODE is mainly produced by 5-LOX, 13-HODE by 15-LOX. *p < 0.05 vs Cy/+ plus lovastatin, **p < 0.01 vs. +/+,***p < 0.001 vs. +/+; n = 5-15.

In comparison to their healthy +/+ counterparts, plasma concentration of 13-HODE, a metabolite produced from LA by 15-lipoxygenase (LOX), was increased in cystic Cy/+ animals (Figure 2A). Plasma concentrations of 9-HODE, a metabolite produced from LA by 5-LOX, however, did not change (Figure 2A). As a result of lovastatin therapy, plasma concentrations of 13-HODE were significantly lower in the treated Cy/+ rats than in the untreated Cy/+ controls (Figure 2A).

In the kidney, the concentrations of 9-HODE as well as 13-HODE were significantly higher in Cy/+ animals (Figure 2B). Lovastatin treatment decreased both LA metabolites to the levels observed in non-cystic +/+ animals. However, this difference was not statistically significant (Figure 2B).

#### Arachidonic acid (AA) metabolism

**Figure 3:**
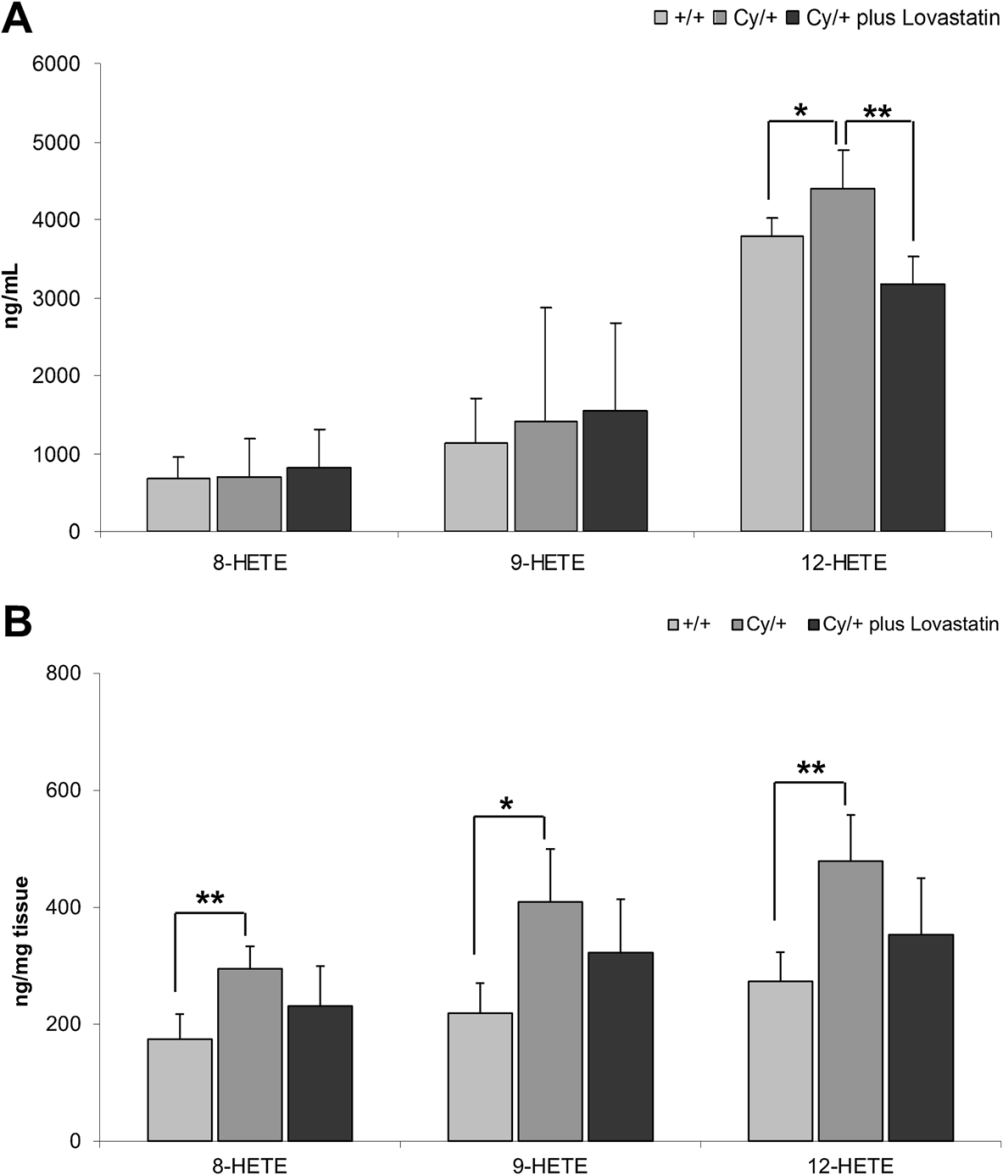
**Changes in pro-inflammatory arachidonic acid metabolites 8-HETE, 9-HETE and 12-HETE in (A) plasma and (B) kidney tissue of healthy (+/+) and cystic Cy/+ rats.** Cy/+ rats were additionally treated with 4 mg/kg/day lovastatin for 5 weeks. 8-HETE and 9-HETE are mainly produced by 8- and 9-LOX, 12-HETE by 12-LOX. Significance levels are given for Cy/+ control versus +/+ control and Cy/+ plus lovastatin versus Cy/+ control: *p < 0.05 vs. +/+, **p < 0.01 vs. Cy/+ lovastatin; n = 5-15.

Plasma and kidney tissue concentrations of 12-HETE, an AA metabolite produced by 12-LOX, was significantly higher in Cy/+ than in +/+ control animals (Figure 3A). While lovastatin treatment was successful in reducing circulating 12-HETE plasma levels, the decrease in the kidney tissue concentrations did not reach statistical significance (Figure 3B).

**Figure 4:**
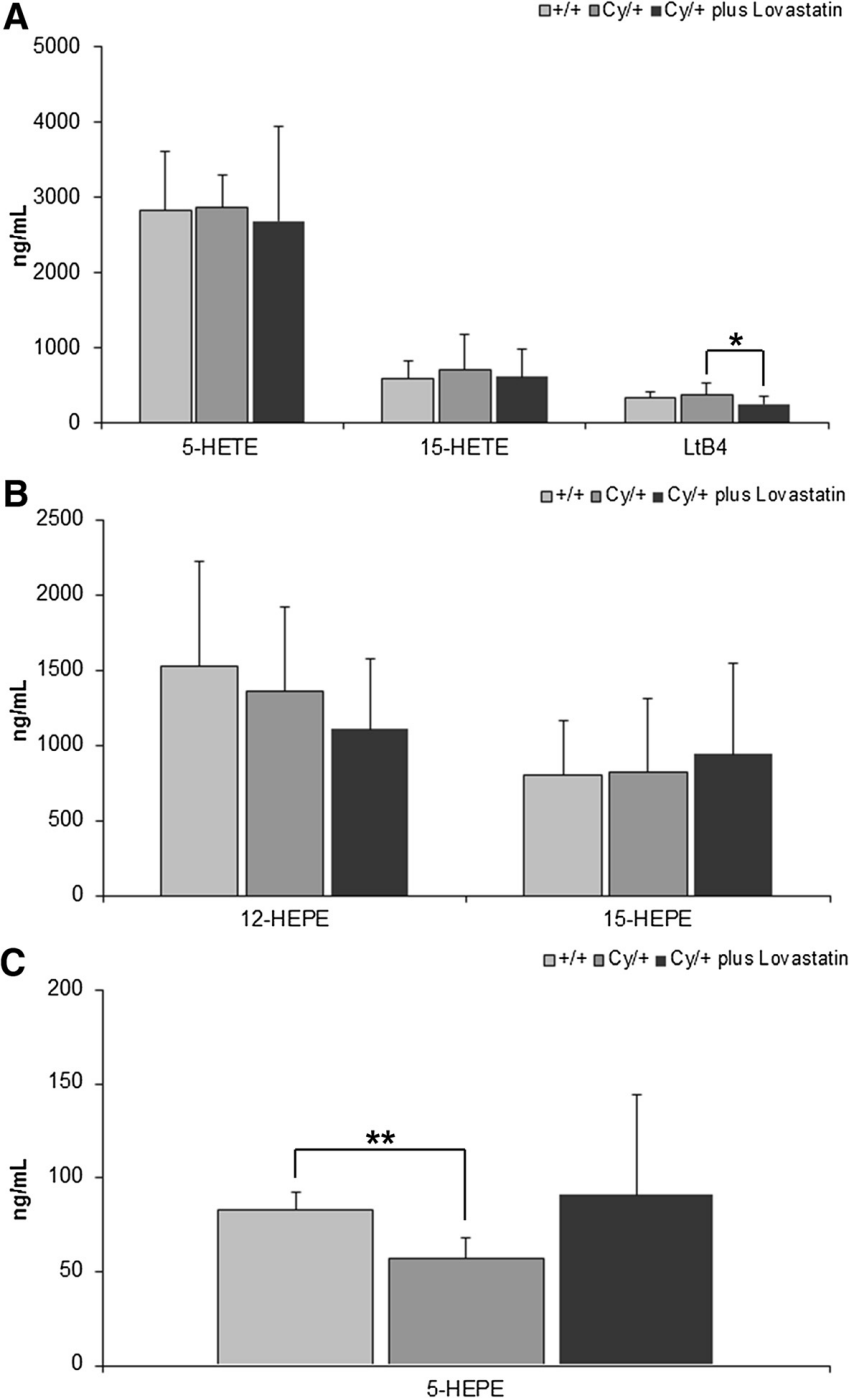
**Changes in pro-inflammatory arachidonic acid metabolites (A) 5-HETE, LtB4 and 15-HETE and (B) potentially anti-inflammatory eicosapentaenoic acid metabolites 12-HEPE, 15-HEPE and (C) 5-HEPE in plasma of healthy (+/+) and cystic Cy/+ rats.** Cy/+ rats were additionally treated with 4 mg/kg/day lovastatin for 5 weeks. Significance levels are given for Cy/+ control versus +/+ control and Cy/+ plus lovastatin versus Cy/+ control: *p < 0.05 vs. Cy/+ plus lovastatin, **p < 0.01 vs. +/+; n = 5-15.

Furthermore, lovastatin was also successful in reducing the plasma concentration of leukotriene B4, a pro-inflammatory product of AA metabolism generated via 5-LOX (Figure 4A). However, no change in the plasma or kidney tissue concentrations of its precursor 5-HETE was observed when comparing cystic versus healthy animals or in Cy/+ animals that received lovastatin therapy (Figure 4A).

In addition, there was a significant difference in the kidney tissue concentrations of 15-HETE between the Cy/+ and control animals (367.8 ± 101.9 ng/mg tissue in Cy/+ animals versus 177.7 ± 36.2 ng/mg tissue in control +/+ animals, p < 0.05, n = 4). This difference was not reflected by 15-HETE plasma concentrations. Lovastatin reduced the kidney tissue concentrations of 15-HETE to 252.4 ± 83.5 ng/mg tissue (not significant vs. vehicle-treatment, n = 5).

In addition to the metabolism via LOX enzymes, AA can be metabolized by cytochrome P450 enzymes and by cyclooxygenases (COX) (Figure 1). However, no significant changes in the kidney concentrations of either 20-HETE (cytochrome P450 metabolite) or prostaglandins (COX metabolite) were noted.

#### Eicosapentaenoic acid (EPA) metabolism

In regards to EPA metabolism, no changes in the concentrations of 12/15-LOX metabolites 12/15-HEPE were observed (Figure 4B). However, Cy/+ rats did show lower plasma levels of anti-inflammatory 5-HEPE as compared to the healthy controls (Figure 4B). Lovastatin increased 5-HEPE plasma concentrations (Figure 4B).

### Endothelial dysfunction markers

**Figure 5:**
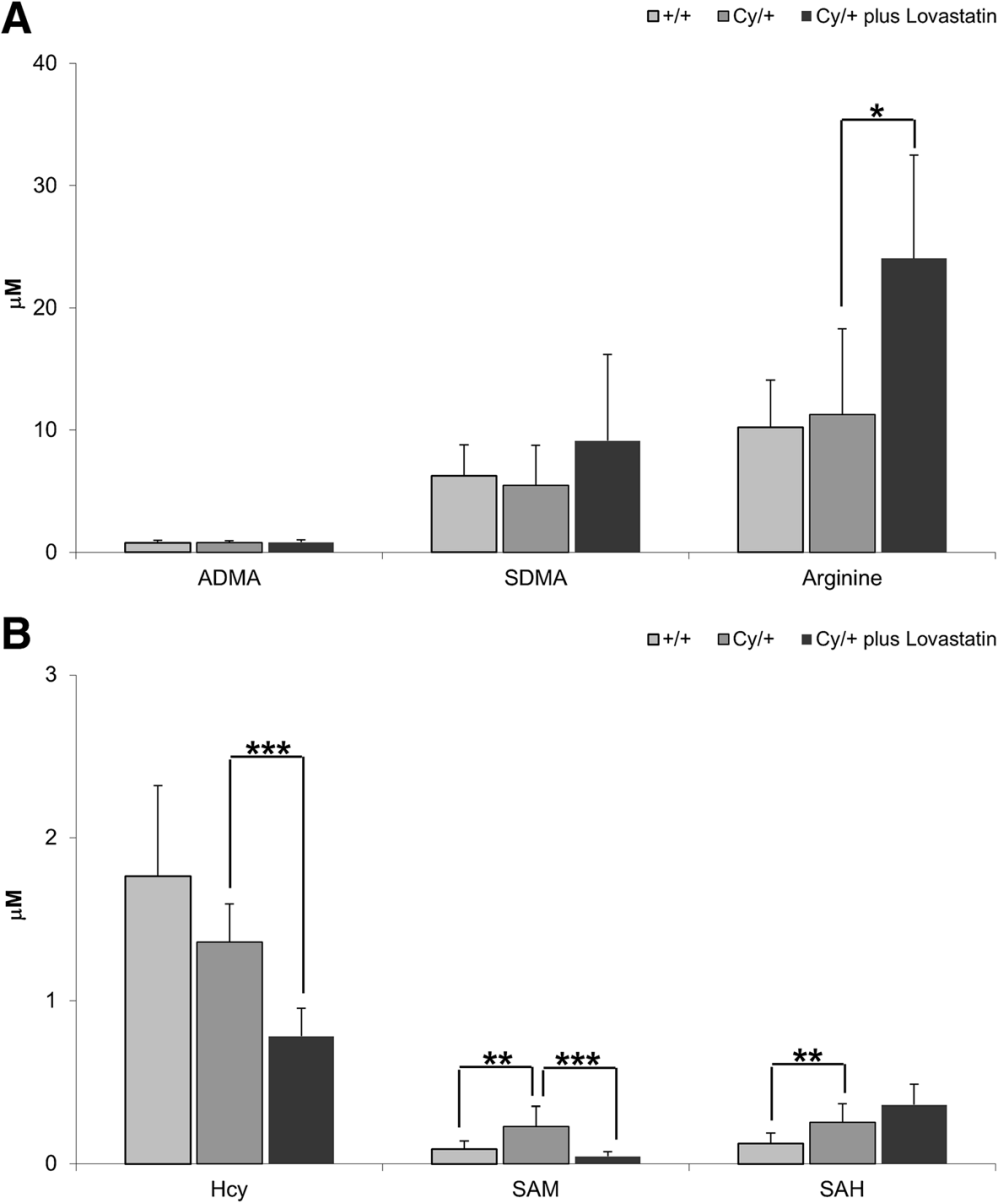
**Changes in endothelial dysfunction markers: (A) NO pathway intermediates arginine, ADMA and SDMA and (B) methionine cycle intermediates HCy, SAM and SAH in healthy (+/+) and cystic (Cy/+) Han:SPRD rats.** Cy/+ rats were additionally treated with 4 mg/kg/day lovastatin for 5 weeks. Significance levels are given for Cy/+ control versus +/+ control and Cy/+ plus lovastatin versus Cy/+ control: *p < 0.05 vs. Cy/+ plus lovastatin, **p < 0.01 vs. Cy/+, ***p < 0.001 vs. Cy/+ plus lovastatin; n = 5-15.

No changes in the plasma concentrations of the endothelial dysfunction markers ADMA and SDMA were noted in either cystic versus healthy animals or following lovastatin treatment (Figure 5A). However, lovastatin was able to increase the concentration of arginine, the positive effector of eNOS, in cystic animals (Figure 5A).

Within the methionine cycle, homocysteine is recycled via methionine into SAM, SAH, and then back to homocysteine. Interestingly, cystic animals showed higher levels of plasma SAM and SAH levels than the +/+ controls, with no change in homocysteine (Figure 5B).

Lovastatin reduced homocysteine as well as SAM plasma concentrations, with interestingly no effect on SAH concentrations (Figure 5B). At the same time in the urine, homocysteine, but not SAM concentrations increased from 24.6 ± 19.5 ng/mg creatinine to 48.5 ± 12.0 ng/mg creatinine (p < 0.05), suggesting an increased excretion rate potentially leading to a reduction of homocysteine plasma levels.

### Kidney glucose metabolism: krebs cycle and glycolysis

The glucose metabolism in the kidneys from Cy/+ animals was significantly altered in comparison to the +/+ controls. The concentrations of Krebs cycle intermediates citrate and succinate were significantly lower in the kidneys of cystic rats (Table 1). This decrease was accompanied by a decrease of kidney glucose concentration (Table 1). Concentrations of aspartate and acetate were also reduced in the kidneys of cystic animals (Table 1). Interestingly, a product of uric acid oxidation and a marker of kidney injury, allantoin, increased almost 3-fold in the Cy/+ rat kidneys (Table 1).

**Table 1:** **Concentration of water-soluble and lipid metabolites as calculated from rat kidney tissue samples using **^**1**^**H-NMR**

**Metabolite**	**+/+**	**Cy/+**	**Cy/+ plus lovastatin**
**Acetate [nmol/g]**	553.6 ± 288.8	229.7 ± 139.3*	272.2 ± 70.5
**Alanine [nmol/g]**	1309.8 ± 300.6	1117.2 ± 412.1	938.3 ± 324.4
**Allantoin [nmol/g]**	623.2 ± 237.7	1572.1 ± 257.1**	1150.1 ± 227.4*
**Aspartate [nmol/g]**	584.0 ± 98.2	381.1 ± 30.5*	572.7 ± 75.9**
**Citrate [nmol/g]**	1366.9 ± 296.8	919.3 ± 150.7*	1419.2 ± 92.2**
**Glucose [nmol/g]**	1145.5 ± 237.9	806.5 ± 113.2*	674.1 ± 104.6*
**Glutamate [nmol/g]**	4615.0 ± 461.6	3669.4 ± 806.1*	3690.8 ± 933.9
**Glutamine [nmol/g]**	1437.1 ± 724.0	1385.9 ± 303.4	1087.4 ± 425.7
**Glycine [nmol/g]**	4763.2 ± 2284.7	3173.1 ± 391.1	4999.2 ± 2408.0
**GPC [nmol/g]**	696.9.3 ± 48.2	541.8 ± 122.2*	631.3 ± 222.5
**3-Hydroxybutyrate [nmol/g]**	442.7 ± 118.8	301.2 ± 43.8*	335.4 ± 97.6
**Lactate [nmol/g]**	4480.7 ± 1303.0	4729.8 ± 696.7	5876.9 ± 393.5*
**Myo-inositol [nmol/g]**	8170.4 ± 1606.9	8789.3 ± 2184.8	9258.2 ± 2478.2
**Succinate [nmol/g]**	3353.4 ± 278.2	2219.6 ± 557.0**	2160.9 ± 420.3
**Taurine [μmol/g]**	12.0 ± 3.7	11.5 ± 1.6	11.7 ± 2.4
**TAG/DAG [μmol/g]**	8.5 ± 2.2	5.1 ± 0.8*	5.9 ± 2.2
**Δ-2 PUFA [μmol/g]**	12.1 ± 2.2	8.0 ± 0.7*	10.0 ± 2.4
**Cholines [μmol/g]**	1.8 ± 0.5	1.3 ± 0.1	1.6 ± 0.2*

Values are presented as means ± standard deviations (n = 4-5). Significance levels are given for Cy/+ versus +/+ animals and Cy/+ plus lovastatin versus Cy/+ animals: *p < 0.05, **p < 0.01; as determined by ANOVA (with post-hoc pairwise multiple comparison Tukey-test). Abbreviations: *DAG*: diacylglycerol, *GPC*: glycerophosphocholine, *PUFA*: polyunsaturated fatty acids, *TAG*: triacylglycerol.

Lovastatin treatment reversed the metabolic changes induced by the PKD in the Cy/+ rats. The citrate concentrations were higher than in the untreated animals and lactate concentrations as well (Table 1), suggesting a recovery of the energy producing Krebs cycle and glycolysis pathways. The increased glucose consumption of the lovastatin-treated kidneys further lowered intra-kidney glucose concentration (Table 1). Allantoin levels were significantly lower as well, but still remained almost 2-fold higher than in the +/+ controls (Table 1).

## Discussion

Patients with ADPKD have an activated pro-inflammatory phenotype [19,20]. The progression of renal disease and hypertension in ADPKD in both children and adults is associated with increases in blood pressure, kidney volume, and cyst volume density [21-24]. Normotensive ADPKD patients with preserved renal function show significantly increased serum levels of vascular inflammatory markers: intercellular adhesion molecule (ICAM)-1, vascular cell adhesion molecule-1 (VCAM-1), P-selectin, E-selectin and soluble Fas (sFas) as compared to healthy controls [25,26]. Furthermore, the inflammation, as indicated by increased serum levels of pro-inflammatory C-reactive protein and IL-8, exhibits a graded relationship with kidney function [27]. Unfortunately, the mechanisms underlying the development of inflammation in ADPKD patients are not fully understood.

Here, we identified elevated plasma concentrations of pro-inflammatory 12/15-LOX metabolites 13-HODE and 12-HETE in cystic Cy/+ versus +/+ rats. Tissue concentrations of the pro-inflammatory linoleic acid LOX metabolites 9-HODE and 13-HODE, the arachidonic acid LOX metabolites 12-HETE as well as 8-HETE and 9-HETE were higher in the kidneys of Cy/+ rats as well. The above described LOX metabolites have also been shown to directly function as key mediators of angiotensin II-induced renin inhibition [28,29] and to contribute to high blood pressure in renovascular hypertension [30].

We have previously shown that treatment with lovastatin decreases kidney weight and cyst volume density in Han:SPRD rats [13]. In the present mechanistic biomarker study, lovastatin was successful in reducing the elevated 13-HODE and 12-HETE plasma levels in Cy/+ animals. In addition, treatment of Cy/+ rats with lovastatin reduced the plasma concentrations of leukotriene B4, possibly through its effect on 5-LOX. 5-LOX-mediated leukotriene B4 generation has been associated with inflammation, athereosclerosis, and vascular disease [31,32] and several cancer types [33]. Interestingly, previous studies have suggested that statins can inhibit the formation of components of the 5-LOX pathway [34-36]. In the kidney, lovastatin was successful in normalizing the levels of pro-inflammatory LOX metabolites as well. On the basis of these observations it can be speculated that a reduction of 12/15-LOX activity should be an attractive target to reduce angiotensin II-mediated oxidative stress, vascular inflammation, cell proliferation and endothelial dysfunction [37-39].

Abnormal vascular function is a feature of ADPKD [40,41]. An increase of ADMA plasma levels in ADPKD patients with normal creatinine clearance has been reported previously [42-44]. This increase is probably associated with the observation that ADPKD patients with normal blood pressures and GFR have substantially decreased acetylcholine-induced endothelium-derived relaxing factor/NO responses and constitutive NOS activity in subcutaneous resistance vessels dissected from biopsy specimens.

In our animal model of PKD, we did not observe any changes in arginine, ADMA or SDMA plasma concentrations between the Cy/+ and +/+ groups. Cystic animals, however, showed elevated plasma concentrations of SAH, that has been reported as an independent risk factor for development and progression of vascular diseases in patients with renal dysfunction [45-47].

Lovastatin elevated plasma arginine, suggesting its beneficial effect on improving the availability of NO [48]. In addition, lovastatin reduced plasma Hcy levels in Cy/+ rats. Since elevated HCy plasma concentrations are regarded as an independent risk factor for development and progression of vascular diseases in patients with renal dysfunction [45-47], lovastatin’s ability to reduce HCy adds to its beneficial effects on the endothelial function.

Previous metabolomics studies showed that kidneys from Cy/+ rats have reduced concentrations of glucose and Krebs cycle intermediates citrate, succinate, and 2-oxoglutarate, as well as of the osmolytes betaine, taurine, and glycerophosphocholine [49]. Our study confirmed these findings of disrupted mitochondrial Krebs cycle activity, and therefore reduced energy supply of the cystic kidney. Lovastatin treatment increased the citrate as well as lactate concentrations in the kidneys of treated Cy/+ rats.

Interestingly, Cy/+ rats showed almost 3-fold higher levels of allantoin, which is formed in rats through the oxidation of uric acid. Lovastatin treatment decreased the allantoin levels in the cystic kidney. Since hyperuricemia has been implicated in the development and progression of chronic kidney disease, and we recently showed that higher serum uric acid levels are associated with earlier onset of hypertension, larger kidney volume and increased hazard for ESRD in ADPKD patients [50], the ability of lovastatin to lower allantoin could represent a potential method for clinical management of hyperuricemia in ADPKD.

## Conclusions

In summary, PKD animals show increased levels of inflammatory bioactive lipid markers derived from the metabolism of arachidonic acid by 5-LOX and 12/15-LOX enzymes. In addition, levels of the endothelial dysfunction marker SAH are increased as well in plasma of PKD animals, and PKD kidneys show a decreased Krebs cycle activity and an increased production of uric acid oxidation product allantoin. Lovastatin decreased measured inflammatory markers, specifically the above mentioned 13-HODE, 12-HETE and leukotriene B4. In addition, lovastatin was successful in reducing the elevated homocysteine and allantoin levels and it also increased plasma arginine, thus positively affecting the NO production and vascular function in cystic animals. In terms of cell metabolism, treatment with lovastatin increased citrate as well as the glycolytical lactate production, thus improving the overall energy state of the cystic kidney. Taken together, our results describe the potential mechanisms of how lovastatin reduces PKD and support the clinical studies of statins that are underway in patients with ADPKD. In addition, the identified pathways could be used as potential therapeutic targets for slowing down the cyst growth.

## Competing interests

The authors declare that they have no competing interests.

## Authors’ contributions

JK carried out the biomarker MS-based biomarker analyses, NMR-based metabolomics and statistical analysis, and drafted the manuscript. IZ carried out the animal studies and sample collections. JK participated in biomarker analyses. ATP and JK participated in the sample preparation and analysis of endothelial dysfunction markers. BYG and RWS participated in the design of the study and helped to draft the manuscript. UC and CLE conceived the study, participated in its design and coordination and helped to draft the manuscript. All authors read and approved the final manuscript.

## Pre-publication history

The pre-publication history for this paper can be accessed here:

http://www.biomedcentral.com/1471-2369/14/165/prepub

## Supplementary Material

Additional file 1 Measurement of endothelial dysfunction markers. Click here for file
