# Supplementary material for: Effects of lovastatin treatment on the metabolic distributions in the Han:SPRD rat model of polycystic kidney disease
Source: BMC Nephrol. 2013 Jul 31;14:165. doi: 10.1186/1471-2369-14-165 (PMC3751441; doi:10.1186/1471-2369-14-165)
Supplement: Additional file 1 — Measurement of endothelial dysfunction markers. [file 1471-2369-14-165-S1.doc]

***Measurement of endothelial dysfunction markers*** Briefly, to 50μL of plasma 25μL of internal standard containing solution (50μM d7-ADMA, d4-cystine, d8-homocystine, d3-methionine and S-methylglytathione, all in HPLC water) and 20μL of 500mM DTT solution were added. For protein precipitation, 200μL of 0.05% trifluoric acid plus 0.1% formic acid containing acetonitrile solution were added to the sample. This solution also contained 500nM d3-SAM and d5-SAH as internal standards for adenosine, SAM and SAH analysis. The sample was finally vortexed for 5 minutes, centrifuged for 10 minutes at 13,000g and transferred into a HPLC vial.

20μL of the supernatant were injected onto a 4.6x12.5 mm guard column (Eclipse XDB-C8, 5μm, Agilent Technologies, Palo Alto, CA) inline with a 3.0x150 mm analytical column (RP-Amide, 3.5μm, Supelco, St. Louis, MI). For adenosine, SAM and SAH analysis, the starting mobile phase concentrations consisted of 5% acetonitrile and 95% 10 mM ammonium formate buffer (pH 3.4) with a flow of 0.6mL/min for the first minute. After one minute, the flow rate was increased to 0.8mL/min and a gradient from 5% to 95% acetonitrile within 2.5 min was run. Acetonitrile was then held at 95% for 0.5 minutes. The column was re-equilibrated for 1 min to starting conditions.

For all other compounds, the gradient started at 3% methanol and 97% 10mM ammonium formate buffer and was maintained at a flow of 0.8mL/min throughout the assay. At minute 4.5, the solvent gradient reached 25% methanol; after this the methanol content was raised to 98% and held for additional 1.5min, after which the columns were re-equilibrated to the starting conditions for the remaining 2 minutes of the assay.
